# Severe Bottleneck Impacted the Genomic Structure of Egg-Eating Cichlids in Lake Victoria

**DOI:** 10.1093/molbev/msae093

**Published:** 2024-05-24

**Authors:** Minami Imamoto, Haruna Nakamura, Mitsuto Aibara, Ryo Hatashima, Ismael A Kimirei, Benedicto B Kashindye, Takehiko Itoh, Masato Nikaido

**Affiliations:** Department of Life Science and Technology, School of Life Science and Technology, Tokyo Institute of Technology, Tokyo, Japan; Department of Life Science and Technology, School of Life Science and Technology, Tokyo Institute of Technology, Tokyo, Japan; Research Center for Integrative Evolutionary Science, The Graduate University for Advanced Studies, SOKENDAI, Kanagawa, Japan; Department of Life Science and Technology, School of Life Science and Technology, Tokyo Institute of Technology, Tokyo, Japan; Department of Life Science and Technology, School of Life Science and Technology, Tokyo Institute of Technology, Tokyo, Japan; Tanzania Fisheries Research Institute (TAFIRI), Dar es Salaam, Tanzania; Tanzania Fisheries Research Institute (TAFIRI), Mwanza, Tanzania; Department of Life Science and Technology, School of Life Science and Technology, Tokyo Institute of Technology, Tokyo, Japan; Department of Life Science and Technology, School of Life Science and Technology, Tokyo Institute of Technology, Tokyo, Japan

**Keywords:** genetic structure, bottleneck, cichlid, paedophage, genetic diversity

## Abstract

Within 15,000 years, the explosive adaptive radiation of haplochromine cichlids in Lake Victoria, East Africa, generated 500 endemic species. In the 1980s, the upsurge of Nile perch, a carnivorous fish artificially introduced to the lake, drove the extinction of more than 200 endemic cichlids. The Nile perch predation particularly harmed piscivorous cichlids, including paedophages, cichlids eat eggs and fries, which is an example of the unique trophic adaptation seen in African cichlids. Here, aiming to investigate past demographic events possibly triggered by the invasion of Nile perch and the subsequent impacts on the genetic structure of cichlids, we conducted large-scale comparative genomics. We discovered evidence of recent bottleneck events in 4 species, including 2 paedophages, which began during the 1970s to 1980s, and population size rebounded during the 1990s to 2000s. The timing of the bottleneck corresponded to the historical records of endemic haplochromines” disappearance and later resurgence, which is likely associated with the introduction of Nile perch by commercial demand to Lake Victoria in the 1950s. Interestingly, among the 4 species that likely experienced bottleneck, *Haplochromis* sp. “matumbi hunter,” a paedophagous cichlid, showed the most severe bottleneck signatures. The components of shared ancestry inferred by ADMIXTURE suggested a high genetic differentiation between matumbi hunter and other species. In contrast, our phylogenetic analyses highly supported the monophyly of the 5 paedophages, consistent with the results of previous studies. We conclude that high genetic differentiation of matumbi hunter occurred due to the loss of shared genetic components among haplochromines in Lake Victoria caused by the recent severe bottleneck.

## Introduction

Invasion of exotic species generally influences the ecomorphologies and demography of endemic species (Salo et al. 2007; Pyšek et al. 2020). The expansion of invaders could be the primary cause of endemic species” population loss, resulting in a drastic change in the population structure and genetic diversity of endemic species (Wang et al. 2005; Fukuda et al. 2016; Burne et al. 2017; Colautti et al. 2017).

Lake Victoria in East Africa suffered a severe environmental upheaval caused by the rapid upsurge of Nile perch (*Lates niloticus*). Nile perch is a large carnivorous fish introduced to Lake Victoria in the 1950s to meet rising commercial demand as a table fish (Fryer 1960; Hauser et al. 1998; Pringle 2005). The primary constituent of the lake's fish fauna is haplochromine cichlids, which experienced explosive adaptive radiation within only 15,000 years, generating 700 endemic species comprising 13 trophic groups (Seehausen 1996; McGee et al. 2020; Meier et al. 2023; Ngoepe et al. 2023). Nile perch expanded their niches in a few decades, and in the 1980s, their catches reached the maximum recorded (Kaufman 1992; Pringle 2005). Meanwhile, haplochromine catches were the worst in that period, and researchers were aware of the loss of their species diversity (Kaufman 1992; Natugonza et al. 2021). Consequently, in the 1990s, more than 200 endemic haplochromines were thought to be extinct after the upsurge of Nile perch, and the population size of each trophic group has also substantially declined (Ribbink 1987; Ogutu-Ohwayo 1990; Kaufman 1992; Witte, Goldschmidt, Wanink, et al. 1992; Seehausen 1996; Natugonza et al. 2021). Although a few decades later, population size has been recovered in some haplochromines (Witte et al. 2007; Kishe-Machumu et al. 2015; Natugonza et al. 2021), the magnitude of population loss in each species and the associated impacts on genetic structure remain to be elucidated.

**Fig. 1.**
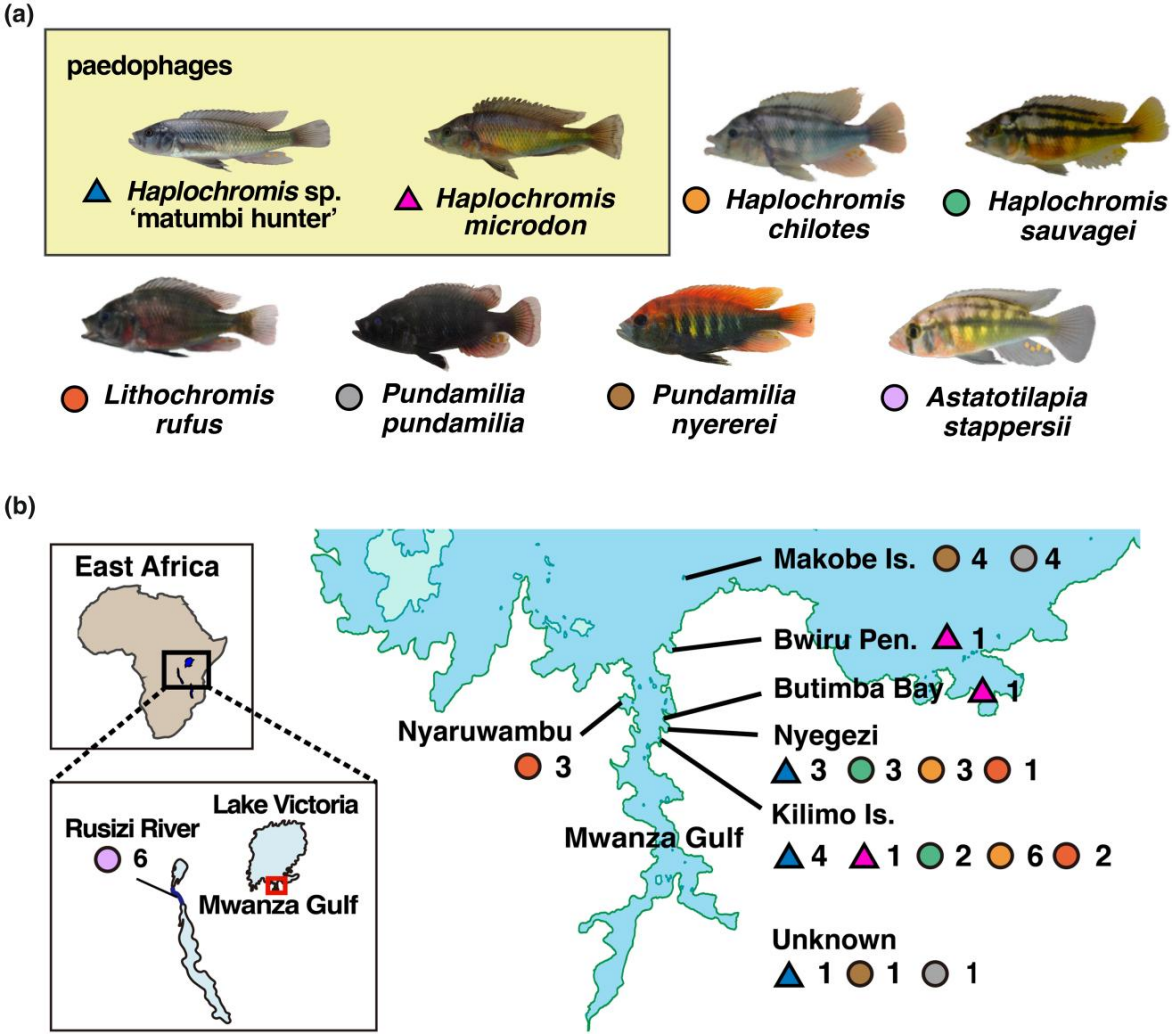
Sampling information and localities of 7 haplochromine cichlids endemic to Lake Victoria and *A. stappersii* as an outgroup. These species were included for genetic statistics comparison. a) Pictures of the 8 species. Colored triangles for paedophages and circles for others next to species names correspond to sampling locations on the map. Paedophages, *Haplochromis* sp. “matumbi hunter,” and *H. microdon* was shaded by a yellow box. A photo of *A. stappersii* was retrieved from Meier, Marques et al. (2017). b) Sampling localities of all samples in a). The area marked by a red square in the bottom left map represents the location of Mwanza Gulf, Lake Victoria, and the map of the enlarged Mwanza Gulf is shown on the right. The number of samples per species obtained in each sampling locality is shown next to markers colored by species, corresponding to the labels in a). Samples without locality information are noted as unknown.

*Haplochromis* sp. “matumbi hunter” (here referred to as matumbi hunter), a haplochromine species found in the Mwanza Gulf belonging to the piscivorous group “paedophage,” has been assumed to have experienced a population bottleneck (Figs. 1a and b). As more than half of the 500 haplochromines in Lake Victoria are not formally described and named (Turner et al. 2001; Witte et al. 2007), matumbi hunter is also a taxonomically undescribed species newly reported by Seehausen (1996). Paedophage is a piscivorous trophic group that eats eggs and fries by stealing them from mouthbrooding cichlids (Greenwood 1959). Along with having typical anatomical features for paedophages, matumbi hunter shows peculiar phenotypes not seen in either other paedophages or rock-dwelling cichlids, such as a slender and gray body (supplementary fig. S1a, Supplementary Material online). Field investigations from the late 1990s to early 2000s suggested a small population size of matumbi hunter, coincided with the era of haplochromine disappearance after the Nile perch invasion (field observation in 2005 by M.A.; Seehausen 1996). Interestingly, while most endemic haplochromines are composed of a mixture of different alleles of V1R2 (olfactory receptor genes), matumbi hunter was fixed to an allele that was exclusively observed for them, implying their poor genetic diversity (Nikaido et al. 2014). Notably, piscivores, including paedophages, were considered the major victims of the Nile perch invasion because their trophic level and feeding substrates (haplochromine cichlids) coincided with Nile perch, resulting in more significant ecomorphological changes than the other trophic groups (Witte, Goldschmidt, Goudswaard et al. 1992; Seehausen 1996; McGee et al. 2015).

This study attempts to understand the evolutionary history of paedophages after the introduction of exotic predators. We first examined the genetic structure of 97 haplochromines endemic to Lake Victoria. We demonstrated that although paedophages share a certain proportion of genetic ancestry, matumbi hunter has a distinct and unique genetic structure. Population genetics statistics and estimated demography in the last few hundred years provided convincing evidence of recent short-term bottleneck events in four species, including the matumbi hunter and the other paedophage *Haplochromis microdon*. The bottleneck period in the four species corresponded to the ages of the mass extinction of haplochromines. We captured the most intensive bottleneck in matumbi hunter, as they showed nearly half of the nucleotide diversity (*π*) compared to others and 99.97% loss of effective population size (*N*_e_) after the bottleneck (decreased from *N*_e_ ≈ 292,785 to *N*_e_ ≈ 90 in 10 generations). Together, the phylogenetic analyses and estimated speciation times confirmed that the matumbi hunter is an ingroup of the paedophage clade. Their distinct genetic structure was caused by their strong bottleneck after the Nile perch expansion.

## Materials and Methods

### Samples and Resequencing

This study used 158 samples of whole genome sequences, including 137 samples sequenced in previous studies obtained from the NCBI database and 21 samples newly re-sequenced in this study. BioSample accession IDs for each sample are available in the “BioSample accession ID” column of supplementary table S1, Supplementary Material online. Wild males of *Haplochromis* sp. “matumbi hunter,” *H*. *microdon*, *H*. *sauvagei*, *H*. *chilotes*, and *L. rufus* were collected in Mwanza Gulf and its surroundings in Lake Victoria by M.A., I.A.K., and B.B.K. Genomic DNA was extracted from either fin clips or muscles following the protocols of the DNeasy Blood & Tissue Kit (QIAGEN) by R.H. After we prepared paired-end libraries following TruSeq DNA PCR Free (350) protocols, whole genome resequencing was performed by Illumina HiSeq X. The whole genome sequence data of 21 newly reported samples have been deposited to the DNA Data Bank of Japan Sequence Read Archive under the BioProject accession ID PRJDB18059.

### Mapping and Variant Calling

Firstly, we generated a master Variant Call Format (VCF) file containing all samples by the following procedures. The quality of short reads was checked by FastQC (Andrews 2010), and adapter sequences and bad-quality reads were removed by fastp (Chen et al. 2018). Raw reads were aligned to the de novo assembled *H. chilotes* (Nakamura et al. 2021) by bwa-mem (v. 0.7.17-r1188) (Li 2013). Unique reads with acceptable mapping quality were kept out using the option “-f 2 -F2052 -q 30' in samtools (v. 1.8) (Li et al. 2009). After extraction of initial variants by bcftools (v. 1.8) (Li 2011), only SNPs with minimum depth = 10, maximum depth = 60, and minimum mapping quality = 60 have remained using the “vcffilter” command in vcflib (Garrison et al. 2022). Single VCF files for all individuals were merged by bcftools. We removed all missing sites, insertions, and deletions, sites that deviate from Hardy–Weinberg Equilibrium (HWE) (*P*-value < 0.001), and non-biallelic sites (using “–max-missing 1,” “–remove-indels,” “–hwe 0.001,” and “–max-alleles 2 –min-alleles 2′ commands in vcftools (v. 0.1.16) (Danecek et al. 2011), respectively).

### Datasets Preparation

We generated the following three VCF datasets from the master VCF file, applying various SNP filtering options to serve specific purposes. The samples included in each dataset are listed in the “ds1,” “ds2,” and “ds3' columns of supplementary table S1, Supplementary Material online.

#### Population Dataset

Kept 155 samples 97 species endemic to Lake Victoria and two ancestral lineages and filtered out SNPs with a frequency below 0.01. To address LD, SNPs with an LD coefficient (*r*^2^) >0.1 were pruned using PLINK (v. 1.9) (Chang et al. 2015) with the “indep-pairwise 50 5 0.1' and “extract” commands, resulting in a final dataset of 1,282,071 SNPs.

#### Statistics Dataset

Seven species from Lake Victoria and *A. stappersii* from the Rusizi River, containing at least three samples per species, were selected to calculate genetic statistics inferring genetic diversity and demography (Fig. 1). The 47 samples were kept from the master VCF file using the command “–keep” in vcftools and remained at 37,335,868 SNPs.

#### Phylogeny Dataset

This dataset included 5 paedophages, 5 sympatric species, and *Astatotilapia burtoni* as an outgroup to reveal the phylogenetic relationship between paedophages and their closely related lineages. We kept 45 samples and excluded SNPs with frequencies <0.03, then converted them to PLINK accessible format (‘–keep,” “–maf 0.03,” and “–plink” commands in vcftools, respectively). SNPs in LD, defined as SNPs with LD coefficient *r*^2^ > 0.1, were estimated and pruned by Plink's “–indep-pairwise 100 5 0.1' and “–extract” commands, respectively, leaving 298,674 SNPs. The dataset for ASTRAL-III was separately prepared by additionally performing haplotype phasing using Beagle (v5.2) (Browning et al. 2021) with 50 iterations after deleting scaffolds with fewer than 50 SNPs to avoid phasing errors, then, the same SNP filtering was applied as mentioned above.

### Genetic Structure and Differentiation Analyses

Using the *1. Population dataset*, the following approaches were used to analyze population structure and differentiation. We performed ADMIXTURE (v1.3.0) (Alexander et al. 2009) to estimate the fraction of components ranging from *K* = 2 to *K* = 8. The PLINK function “–pca” was used to create eigenvalues and vectors. Pairwise *F*_ST_ (Weir and Cockerham 1984) between 8 species were calculated in 10 kb sliding windows by vcftools “–fst-window-size 10,000 –weir-fst-pop” command using *2. Statistics dataset*.

### Summary Statistics

Using *2. Statistics dataset*, we calculated nucleotide diversity (*π*) and Tajima's *D* within a species in a 10 kb window using the vcftools functions “–window-pi 10,000' and “–TajimaD,” respectively. The inbreeding coefficient was computed using the “–het” command. LD coefficients (*r*^2^) between SNPs located within 10 kb were observed using PopLDdecay (Zhang et al. 2019). Additionally, we prepared a filtered VCF dataset for ROH analysis by removing SNPs with a frequency <0.03 and LD coefficients (*r*^2^) >0.1 using the “–indep-pairwise 5,050.1' PLINK command. The SROH and NROH within an individual, using a homozygous region lasting more than 150 kb, were inferred using PLINK commands “–homozyg –homozyg-density 5 –homozyg kb 150 –homozyg-snp 2'.

### Effective Population Size

We ran GONE (v1.0) (Santiago et al. 2020) to estimate demography in the last few hundred years. We trimmed *2. Statistics dataset* by retaining scaffolds with more than 150,000 SNPs. To prevent biased estimates in admixed populations, we separately gave the greatest value of recombination (0.05 for matumbi hunter, *H. microdon*, and *H. sauvagei*, and 0.01 for *H. chilotes*, *P. pundamilia*, *P. nyererei*, *L. rufus*, and *A. stappersii*). The effective population size in the previous 700 generations was determined by randomly extracting 22,000 SNPs per scaffold (specified as “NGEN = 700,” “NBIN = 700,” maxNCHROM = −99,” and “maxNSNP = 22,000' in INPUT_PARAMETERS_FILE), replicating 200 times to infer the consensus demography.

### Phylogenetic Relationships

We constructed phylogenetic trees by running 5 different software programs below using the *3. Phylogeny dataset*, which was tailored by applying proper SNP filtering, including LD pruning.

#### *RAxML-NG, IQ-TREE2*, and *SVDquartets*

vcf2phylip were used to convert a phylogeny dataset to a phylip format (Ortiz 2019). ModelTest-NG (v. x.y.z.) (Flouri et al. 2015; Darriba et al. 2020) chose General Time Reversible (GTR) + G4 as a suitable model. RAxML-NG (v. 0.9.0) (Kozlov et al. 2019) was used to estimate the ML tree with 200 bootstrap repetitions. IQ-TREE2 (v. 2.0.3) (Nguyen et al. 2015) has been performed with 1,000 ultrafast bootstrap replicates (Hoang et al. 2018) and the Transversion model equal base freq (TVMe) + R5 model estimated by ModelFinder (Kalyaanamoorthy et al. 2017). Inferring the phylogenetic tree using SVDquartets, *3. Phylogeny dataset* was transformed to nexus-format by convert_vcf_to_nexus.rb (available at https://github.com/mmatschiner/tutorials/blob/master/species_tree_inference_with_snp_data/src). To infer the multispecies coalescent tree, we ran SVDquartets (Chifman and Kubatko 2014, 2015), an implemented package in PAUP (v 4.0) (Swofford 2003).

#### ASTRAL-III

As mentioned in “Dataset preparation,” we used a phased-version of *3. Phylogeny dataset* to construct a tree by ASTRAL-III. Running raxml_sliding_window.py (available at https://github.com/simonhmartin/genomics_general) produced a multi-tree file containing 7,980 gene trees in a 20 kb sliding window with a 5 kb stepwise. Finally, we ran ASTRAL-III (v5.7.8) (Zhang et al. 2018) with the default parameters.

#### SNAPP

The SNAPP program has been executed using the BEAST 2.0 platform (Bryant et al. 2012; Bouckaert et al. 2019). As SNAPP requires significant computing power to run with large datasets, we replicated the analysis by generating 10 thinned datasets containing randomly selected 1,000 SNPs from LD-pruned *3. Phylogenetic datasets*. Thinned datasets were converted to nexus-format by vcf2phylip. XML files for the BEAST 2.0 run were manually generated in BEAUTi. We ran SNAPP analysis for 2 million generations, recording topologies every 1,000 steps. The effective sample size of each run exceeded 200, which was checked by Tracer (Rambaut et al. 2018). Outputs were visualized in 3 different ways: independently annotated trees (un-concatenated, supplementary fig. S5, Supplementary Material online), a DensiTree of concatenated multi-trees (Fig. 4c), and its consensus tree (Fig. 4d). Un-concatenated trees were independently visualized in layers by DensiTree (Bouckaert and Heled). Next, the concatenated multi-trees were generated by merging 10 un-concatenated trees in LogCombiner and drawn as layered trees by DensiTree. Finally, we obtained the consensus tree from concatenated multi-trees by TreeAnnotator with 10% burn-in and drawn in FigTree (v1.4.4) (Rambaut 2018).

### Topology Weighting Across the Genome Using TWISST

Topology weighting was performed by TWISST (Martin and Van Belleghem 2017). We used raxml_sliding_window.py to construct three multiple-tree files comprising 731,216, 366,759, and 125,123 gene trees in 50, 100, and 150 SNP windows from the phased-VCF. We defined *A. burtoni* as an outgroup, divided the remaining ten species into 4 groups based on previously estimated phylogenies (Fig. 5a), and performed topology weightings for 15 different topologies.

### Speciation Time

Speciation times for all possible 21 pairs in 7 species were calculated by SMC++ (v1.15.4) (Terhorst et al. 2017). *H. microdon* was excluded from this analysis because we were concerned that a small sample size might disturb an inference of the split time. A VCF dataset prepared in the previous GONE analysis was converted into “.smc” format (‘smcpp vcf2smc” command in SMC++). We generated 100 inputs per scaffold for replicated runs by bootstrap_smcpp.py (available at https://github.com/popgenmethods/smcpp/issues/37), running *N*_e_ estimations for 100 replicated runs (with the command “smcpp estimate’), setting the per-generation mutation rate as 3.5 × 10^−9^ (Malinsky et al. 2018). We estimated speciation time with the function “smcpp split” in SMC++, setting the per-generation mutation rate as 3.5 × 10^−9^ (Malinsky et al. 2018).

## Results

### The Distinct Genetic Structure of Matumbi Hunter

**Fig. 2.**
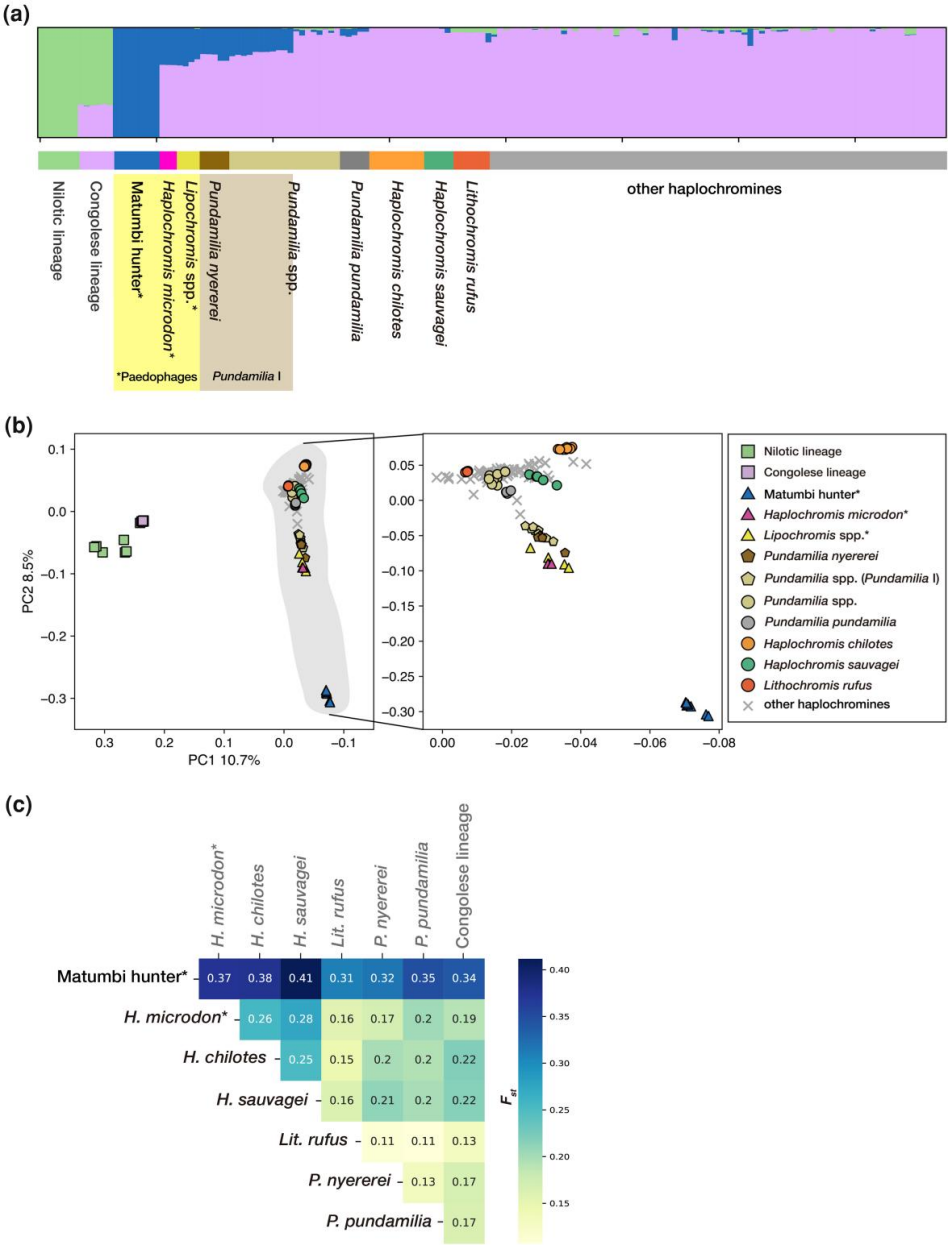
Distinct genetic structure and the signs of a severe bottleneck for matumbi hunter. The Nilotic lineage (*A. bloyeti*, *A. paludinosa*, *H. gracilior*, and *T. pharyngalis*) and the Congolese lineage (*A. stappersii*) are added as an outgroup. Paedophages, matumbi hunter, *H*. *microdon*, and *Lipochromis* spp. (*L. parvidens*, *Lip. melanopterus*, and *L. cryptodon*) have asterisks on each name label. a) ADMIXTURE (*K* = 3) for 97 Lake Victoria haplochromines and outgroup lineages. Paedophages are shaded by a yellow box. Species belonging to “*Pundamilia* I,” *P. nyererei*, and five species labeled as part of “*Pundamilia* spp.” (*P*. sp. “big blue red,” *P*. *igneopinnis*, *P*. sp. “nyererei-like,” *P*. sp. “orange,” and *P*. sp. “pundamilia-like’), are shaded by a light brown box. Labels for all samples are listed in the column “Label in ADMIXTURE” in supplementary table S1, Supplementary Material online. b) Principal Component Analysis (PCA) for 97 Lake Victoria haplochromines with 2 ancestral lineages (square markers) as an outgroup (left) and without an outgroup (right). Haplochromines endemic to Lake Victoria are shaded by gray in the left figure. The PC1 axis is transposed in descending order for graphical purposes for both figures. The contribution rate for each principal component is written on the axis. “Paedophages’ and “*Pundamilia* I” are plotted as triangle and pentagon markers, respectively. Species belonging to “*Pundamilia* I,” except for *P. nyererei*, are plotted as light brown pentagon markers. c) A heatmap of pairwise weighted *F*_ST_ (*x*-axis vs. *y*-axis) in a total of 28 pairs for 8 species. *F*_ST_ for the relevant pair is written in a box; for example, the *F*_ST_ between matumbi hunter and *H*. *microdon* is 0.37.

First, we performed an ADMIXTURE for 97 endemic haplochromines and 2 ancestral lineages to compare the shared genetic components among species (Fig. 2a and supplementary fig. S1, Supplementary Material online). The Nilotic lineage (*Astatotilapia bloyeti*, *A. paludinosa*, *Haplochromis gracilior*, and *Thoracochromis pharyngalis*) and the Congolese lineage (*Astatotilapia stappersii*), were included in the analysis as an outgroup. For the 8 species in Fig. 1a, at least 3 genome samples per species were used for the analysis, and the rest were composed of 1 genome per species.

We observed the lowest cross-validation error rate for *K* = 2 (upplementary fig. S1b, Supplementary Material online), reflecting the young age of radiation and ongoing introgressions of Lake Victoria haplochromines (Meier, Sousa, et al. 2017). Just having 2 different components (*K* = 2) made it difficult to detect species-specific genetic backgrounds for endemic haplochromines (supplementary fig. S1a, Supplementary Material online). To have more resolution in shared genetic components among species, we used *K* = 3 (Fig. 2a). Because the Congolese and Nilotic lineages diverged earlier, in *K* = 3, we expected to see three components reflecting the genetic background of each drainage system. However, we captured the following three components: (i) green for the 2 ancestral lineages; (ii) pink for the Congolese lineage and all Lake Victoria species except matumbi hunter; and (iii) blue for paedophages (matumbi hunter, *H*. *microdon*, and *Lipochromis* spp.) and some *Pundamilia* species (Fig. 2a). Although matumbi hunter was solely composed of blue, a large proportion of endemic species were mostly composed of pink, suggesting that matumbi hunter is genetically distinct from other haplochromines. However, in larger *K*, other paedophages (*H*. *microdon* and *Lipochromis* spp.) carried components that matumbi hunter also had, reflecting the genus-level shared ancestry (supplementary fig. S1, Supplementary Material online a). *Pundamilia nyererei* and 5 species were labeled as part of “*Pundamilia* spp.” (*P*. sp. “big blue red,” *P*. *igneopinnis*, *P*. sp. “nyererei-like,” *P*. sp. “orange,” and *P*. sp. “pundamilia-like’), also shared components with paedophages in *K* = 2 to 5 (shaded by a light brown box with the “*Pundamilia* I” label in Fig. 2a and supplementary fig. S1a, Supplementary Material online). These species were previously classified as “*Pundamilia* I,” a taxonomic group that was the sister lineage of paedophages (Meier et al. 2023).

We also performed Principal Component Analysis (PCA) with the same dataset as used in ADMIXTURE (Fig. 2b and supplementary fig. S2, Supplementary Material online). PC1 represented genetic differences by drainage systems, separating samples by the Nilotic, Congolese, and Lake Victoria lineages, whereas PC2 separated matumbi hunter and others, suggesting that matumbi hunter cluster could be separated from the Lake Victoria cluster in which other paedophages are included.

Next, we measured the pairwise *F*_ST_ for the 8 species (Fig. 2c). Notably, the matumbi hunter exhibited higher *F*_ST_ values ranging from 0.31 to 0.41, including an *F*_ST_ of 0.37, with the other paedophage *H. microdon*. *F*_ST_ for pairs of Lake Victoria cichlids except for matumbi hunter ranged from 0.11 to 0.28 (Fig. 2c). Generally, a high *F*_ST_ indicates that pairs are distantly related. However, low genetic diversity after bottleneck also increases *F*_ST_, although species are closely related (Chakraborty and Nei 1974; Chakraborty and Nei 1977). Considering the previously inferred small population size (Seehausen 1996) and poor genetic diversity (Nikaido et al. 2014) of matumbi hunter, we hypothesized that the strong bottleneck might explain their increased *F*_ST_.

### Signatures of the Recent, Short-term, and Strongest Bottleneck in Four Endemic Haplochromines, Including two Paedophages

**Fig. 3.**
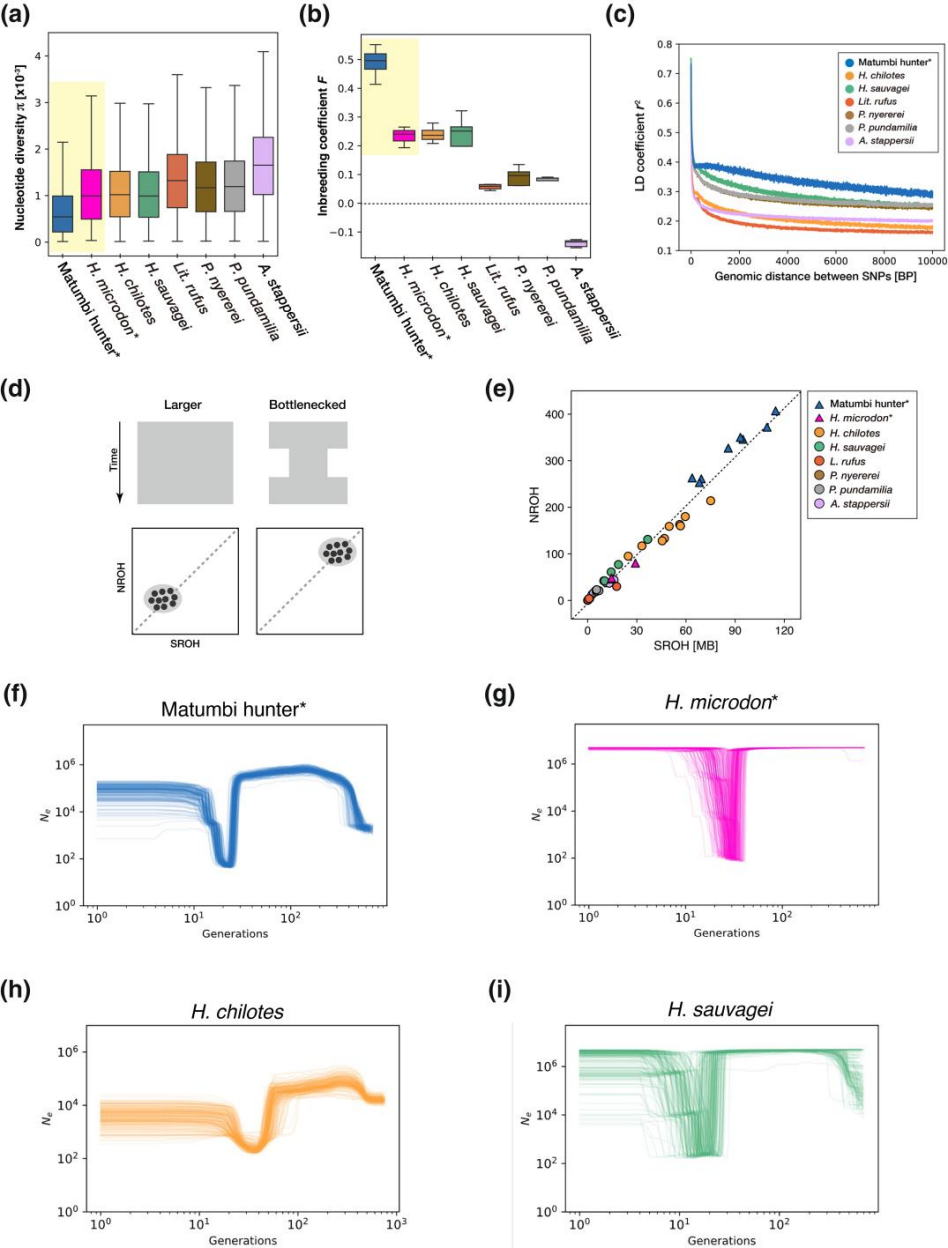
Estimated population genetics statistics and demography of haplochromine species. Paedophages (matumbi hunter and *H. microdon*) are either shaded by a yellow box or have an asterisk on the name label. a) 10 kb windowed nucleotide diversity (*π*) across a genome calculated by species. b) Average inbreeding coefficient (*F*) by species. We calculated *F* for each sample and then averaged them by species. c) Comparison of LD decay, plotting LD coefficient (*r*^2^) of Single Nucleotide Polymorphisms (SNPs) having a distance within 10 kb. *H.* microdon has been excluded from this analysis because an insufficient number of samples per species may cause a bias in estimating *r*^2^. d) Expected correlation between the SROH and the total NROH under certain demographic histories [larger (left) and bottlenecked populations (right)]. Theoretically, a short and small number of ROH regions (both SROH and NROH are smaller) will be detected in a large and expanded population, whereas long and large numbers of ROH (both SROH and NROH are larger) can be observed in a small and bottlenecked population (Ceballos et al. 2018). e) Observed correlation of SROH and NROH. Each plot corresponds to one sample. Homozygous regions lasting more than 150 kb were defined as ROH regions. Some samples showed close values of SROH and NROH, thus plots were overlapped. f and g) Effective population size (*N*_e_) of 4 bottlenecked species [f) matumbi hunter, g) *H*. *microdon*, h) *H. chilotes*, and i) *H*. *sauvagei*] in the past 700 generations. The GONE estimate was repeated 200 times, and all results were plotted.

Based on the results of the above analyses, we regard matumbi hunter as a proper candidate to investigate an invader-driven bottleneck. We calculated genetic statistics and estimated demographic patterns for the 8 species shown in Fig. 1. First, we calculated the genome-wide nucleotide diversity (*π*, the mean pairwise differences per base pair) in a 10 kb nonoverlapping window (Fig. 3a and supplementary table S2, Supplementary Material online). Two paedophages (matumbi hunter and *H. microdon*), *Haplochromis chilotes*, and *H. sauvagei* demonstrated lower nucleotide diversity among the eight species. However, the average nucleotide diversity of matumbi hunters was about half of the other three species. Corresponding to the low nucleotide diversity values indicating a decrease in population size, their inbreeding coefficient (*F*) was high (Fig. 3b and supplementary table S2, Supplementary Material online). This result implies that mating between closely related individuals frequently occurred, resulting in an excess number of homozygous sites. These species also exhibited relatively high *F*_ST,_ possibly due to bottleneck events (Fig. 2c). We also estimated the decay of pairwise linkage disequilibrium (LD) by comparing the squared correlation coefficient *r*^2^ of pairwise Single Nucleotide Polymorphisms (SNPs) located at a distance <10 kb (Fig. 3c and supplementary table S2, Supplementary Material online). Although *H. microdon* was not included in this analysis due to a small sample size and the *r*^2^ of *H. chilotes* was lower than that of *P. nyererei* and *P. pundamilia*, increased *r*^2^ in distantly located SNPs observed for matumbi hunter and *H. sauvagei*, indicating that recombination and novel mutations have been less accumulated due to the recent bottleneck (Fig. 3c).

We measured genome-wide runs of homozygosity (ROH, continuous homozygous blocks in the genome), which we used to infer holistic demographic events by finding a correlation between the sum total length of ROH (SROH) and the total number of ROH (NROH) (Ceballos et al. 2018). Matumbi hunter showed prolonged SROH and excessive NROH, consistent with a typical ROH pattern for a bottlenecked population, further supporting a previous bottleneck event (Figs. 3d and e). Additionally, SROHs and NROHs of *H. microdon*, *H. chilotes*, and *H. sauvagei* were mostly high.

Tajima's *D*, a genetic statistic used to infer demographic events such as bottleneck (Tajima 1989), was also estimated (supplementary fig. S3, Supplementary Material online and supplementary table S2, Supplementary Material online). The mean of the distribution of Tajima's *D* would be 0 in a population maintaining a constant population size under neutrality, while a positive value indicates a past bottleneck event (Tajima 1989; Schmidt and Pool 2002). However, the genome-wide mean Tajima's *D* in bottlenecked species was not significantly greater than zero except for *H. chilotes*, which showed positive Tajima's *D* (supplementary fig. S3, Supplementary Material online and supplementary table S2, Supplementary Material online).

Additionally, we inferred the changes in effective population size (*N*_e_) within the last 700 generations from the GONE software (Figs. 3f to i and supplementary fig. S4, Supplementary Material online). This study assumed the cichlid generation time to be 1 year, according to observations in our laboratory, including wild-type strains. Again, we detected bottleneck events in the four species as observed by the genetic statistics comparison (Figs. 3f-i). Although the exact timing of the bottleneck was different, its demography was similar: the population size started to decrease 40 to 30 generations ago and then rebounded from 20 to 10 generations ago. Since most of our samples were collected in 2018, their bottleneck began in the 1970s to 1980s and the small population size remained for 10 to 20 years, with the following population expansion in the 1990s and 2000s. In contrast, the other four species examined (*P. nyererei*, *P. pundamilia*, *Lithochromis rufus*, and *A. stappersii*) did not show a recent short-time bottleneck and quick recovery of population size, while they also appear to have experienced a decrease in population size within recent 10 years (supplementary fig. S4, Supplementary Material online).

These findings suggest that the 4 species that inhabit Mwanza Gulf, the southern part of the lake (matumbi hunter, *H. microdon*, *H. sauvagei*, and *H. chilotes*) experienced a population bottleneck, although the impact on paedophages appears relatively higher. Importantly, we would like to emphasize that the matumbi hunter underwent the strongest bottleneck in any other species, which was shown by all statistics and demography estimation ([the lowest nucleotide diversity, highest homozygosity, elevated LD decay, and the smallest *N*_e_ in the bottleneck period (*N*_e_ ≈ 90, 20 generations ago), except for Tajima's *D* (Figs. 3a to f, supplementary fig. S3, Supplementary Material online and supplementary table S3, Supplementary Material online)].

### Phylogenetic Trees Inferred by Using the Five Methods Supported the Monophyly of Paedophages

**Fig. 4.**
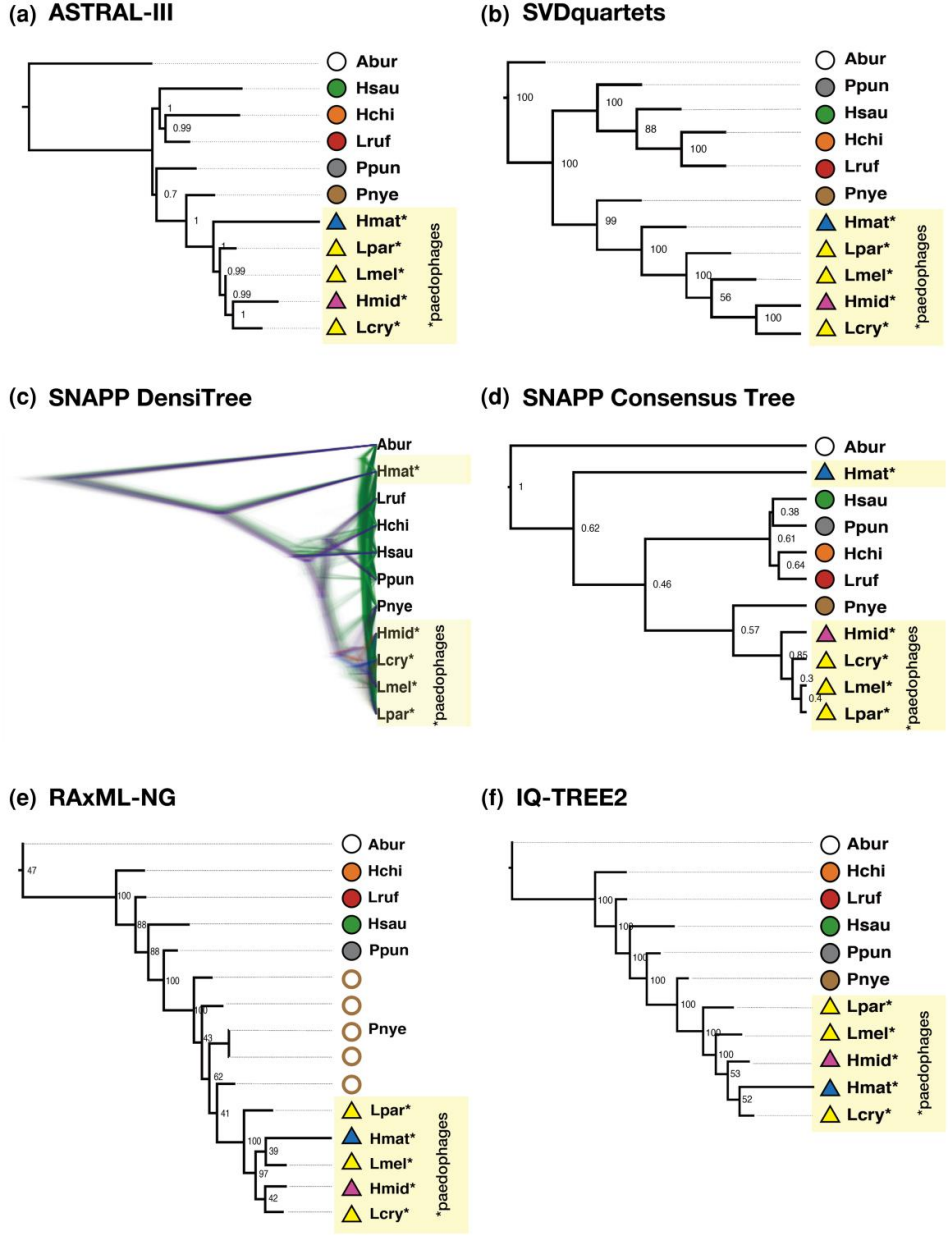
Phylogenies estimated by 4 different methods. Monophyly of paedophages was highly supported by phylogenies estimated by all the methods except for SNAPP. All the trees were rooted on *A. burtoni* (Abur), a riverine species. Samples are labeled with species name abbreviations: Hmat (matumbi hunter), Hmid (*H. microdon*), Lpar (*L. parvidens*), Lmel (*Lip. melanopterus*), Lcry (*L. cryptodon*), Pnye (*P. nyererei*), Ppun (*P. pundamilia*), Hchi (*H. chilotes*), Hsau (*H. sauvagei*), and Lruf (*L. rufus*). Paedophages are indicated as triangles shared by yellow boxes with asterisks on the name labels. Bootstrap values and posterior probabilities for each node are shown in b), e), f), and a), d), respectively. In b), e), and f), samples that formed monophyletic clades by species were collapsed for graphical purposes, except for *P. nyererei* (Pnye) in e), which showed a paraphyletic topology (brown outlined white circle). a) A consensus phylogenetic tree obtained by ASTRAL-III estimated from 7,980 gene trees in a 20 kb sliding window with a 5 kb overlap. b) Coalescent tree built by SVDquartets with 100 bootstrap replicates. Note that SVDquartets can estimate only the topology but not the branch length. c) Layered DensiTree drawn from concatenated SNAPP multi-trees and its maximum clade credibility tree d). In DensiTree, the more commonly observed trees are colored in the order of blue, red, and green, while the rest are dark green, respectively. e) The ML tree estimated by RAxML-NG with 200 bootstrap repetitions under the General Time Reversible (GTR) + G4 model. f) The ML tree estimated by IQ-TREE2 with 1,000 ultrafast bootstrap replicates under the Transversion model equal base freq (TVMe) + R5 model.

Subsequently, we constructed phylogenetic trees using multiple genomic data per species using 5 different methods: 3 coalescence-based methods (Figs. 4a to d) and 2 maximum-likelihood (ML) methods (Figs. 4e and f). The monophyly of 5 paedophages [matumbi hunter (Hmat), *H. microdon* (Hmid), *Lipochromis parvidens* (Lpar), *L. melanopterus* (Lmel), and *L. cryptodon* (Lcry)] was highly supported in all estimated trees except for the SNP and AFLP Package for Phylogenetic analysis (SNAPP) consensus tree (Fig. 4d). For the SNAPP analysis, we independently generated 10 datasets composed of 1,000 randomly sampled SNPs and concatenated them to estimate the maximum clade credibility tree as the consensus tree. In the SNAPP consensus tree, matumbi hunter was located at the basal position in the Lake Victoria clade (Fig. 4d). The trees with higher posterior probabilities (blue colored) demonstrated in Fig. 4c mostly corresponded to the consensus tree. However, the topologies of the other trees differed from those of the consensus tree (Fig. 4c and d). Similarly, in separately imaged DensiTrees of un-concatenated datasets, matumbi hunter was included in the paedophage clade in 7 of 10 topologies (supplementary fig. S5, Supplementary Material online). Therefore, they likely form a distinct phylogenetic group as a dietary group.

**Fig. 5.**
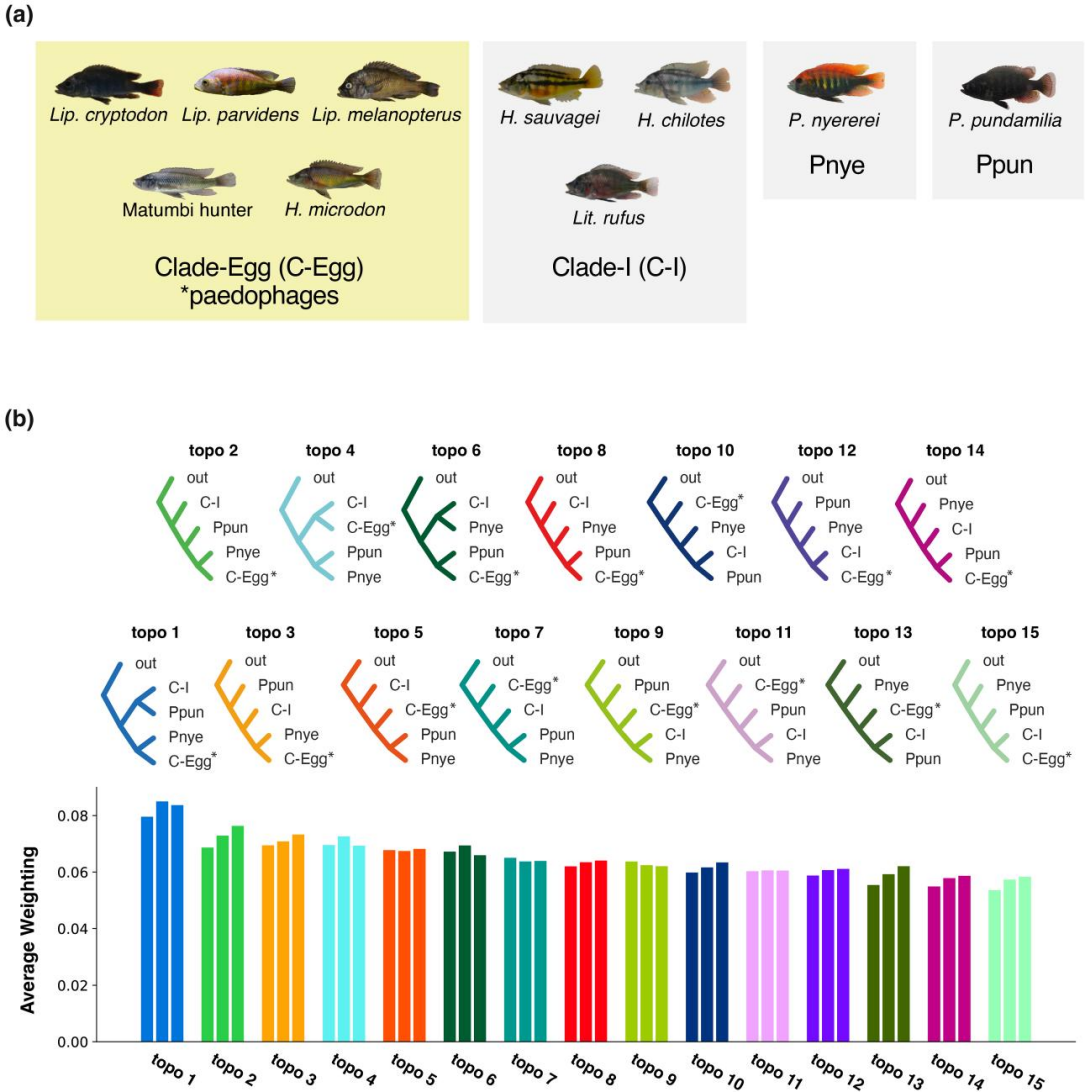
Frequently observed topologies in TWISST matched with the trees built by phylogenetic analyses. a) Groupings used for TWISST analysis. We defined 4 topological groups: 5 paedophages as C-Egg; 3 species often forming monophyletic clades as Clade-I; *P. nyererei*; and *P. pundamilia*. b) The 15 possible topologies for the 4 groups defined in a) and their average weightings (the frequency of a certain topology constructed in a SNP window) in 3 window sizes. *A. burtoni* was set as an outgroup (out). An asterisk indicates paedophages (C-Egg). Three bars for each topology, represented by a unique color, are average weightings observed in the 50 (left), 100 (center), and 300 (right) SNP windows. Bars are shown in descending order of average weightings among the 3 windows.

To assess the plausibility of the estimated phylogenetic relationships in Fig. 4, we performed TWISST to estimate the most frequently observed tree across the genome. We defined four groups that often formed a monophyletic clade in the phylogenetic analyses (Fig. 5a). We calculated the average weighting per topology, i.e. the average frequency that the topology appeared in the estimated phylogeny for SNP windows, for all the possible 15 topologies for the 4 groups. Topo 1 ([out, {C–I, Ppun}, {Pnye, C-Egg}]); was the most frequently observed topology in all SNP window sizes (50, 100, and 150 SNPs), matching with the topology estimated by SVDquartets (Figs. 4b and 5b). The three topologies that appeared most frequently (topo1, topo2, and topo3) supported the sister relationship of *P. nyererei* (Pnye) and the paedophages (C-Egg), which were also estimated as sister clades in all inferred phylogenetic trees in Fig. 4. The close phylogenetic relationship between *P. nyererei* and paedophages corresponds to a similar extent of genetic components shared among them, as shown in the ADMIXTURE analysis mentioned earlier (Fig. 2a and supplementary S1, Supplementary Material online).

**Fig. 6.**
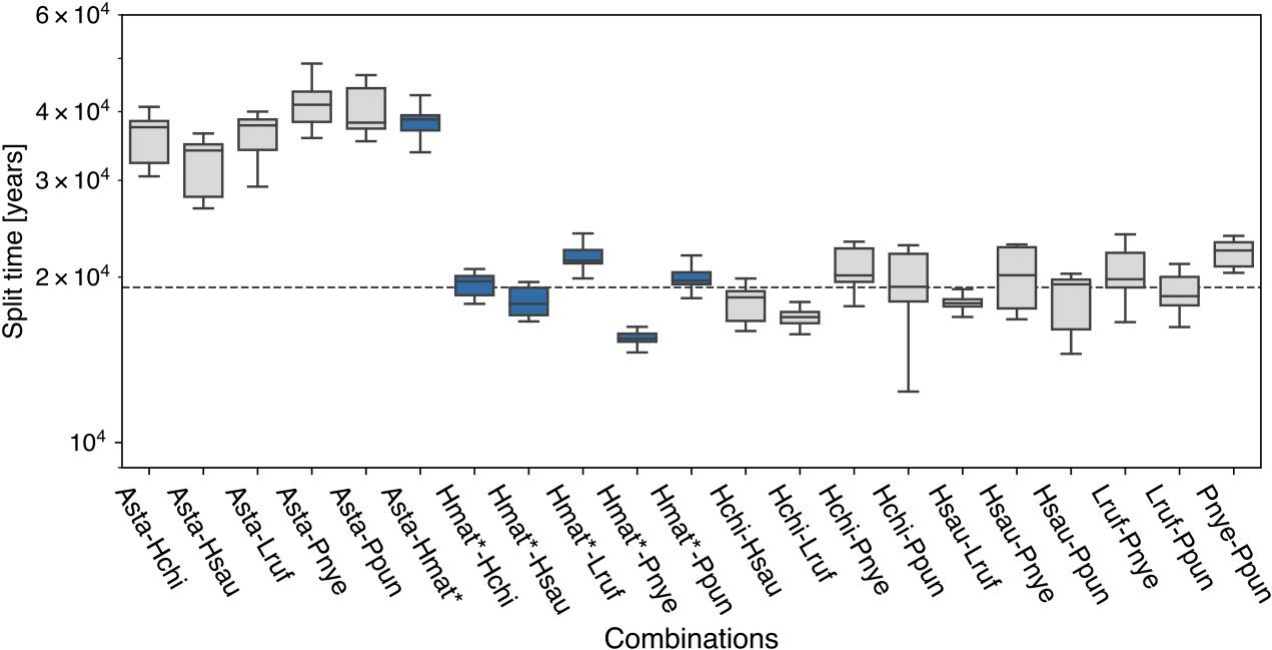
Split times of pairs of the 7 species indicated the codiversification of Lake Victoria haplochromines. Samples are labeled with species name abbreviations: Asta (*A. stappersii*), Hmat (matumbi hunter), Hchi (*H. chilotes*), Hsau (*H. sauvagei*), Lruf (*L. rufus*), Pnye (*P. nyererei*), and Ppun (*P. pundamilia*). Paedophages have an asterisk on the name label. The estimation of the split time was repeated 100 times per pair. The per-generation mutation rate was defined as 3.5 × 10^−9^, referring to Malinsky et al. (2018). The mean split time among pairs of Lake Victoria cichlids (19,158 yr) was drawn as a dashed line. Split times in pairs of matumbi hunter are colored blue.

We also estimated the split times for 21 combinations in 7 species to clarify whether the elevated genetic differentiation between species pairs, particularly including matumbi hunter, was not due to their old divergence (Fig. 6). The average split time between *A. stappersii*, a riverine species, and matumbi hunter was ∼40,000 yr, corresponding to the range of the average split time between *A. stappersii* and other haplochromines of Lake Victoria. Additionally, the average split times among Lake Victoria's haplochromines were all approximately 19,000 yr, corresponding to the haplochromines’ radiation in Lake Victoria (Johnson et al. 2000; Meier, Marques, et al. 2017; Meier et al. 2023).

## Discussion

This study discovered multiple evidence of bottleneck events in Lake Victoria haplochromines by genome analyses. First, we elucidated the genetic structure of endemic haplochromines and shared ancestry among the genera. The population genetics statistics and past demography were then estimated to investigate the previous bottleneck events possibly triggered by an ecological collapse after the expansion of the Nile perch. We constructed phylogenetic trees by multiple methods using genome-wide SNPs extracted from representative species with multiple samples. Constructed phylogenetic trees revealed the monophyly of paedophages, one of the piscivorous haplochromines, and their close phylogenetic relationship with *P. nyererei*, corresponding with previous phylogenetic studies. This study shows the extent of the population bottleneck and its genetic consequences for paedophage, one of the piscivorous haplochromines in Lake Victoria.

### Evidence of Bottleneck Events Following the Upsurge of Nile Perch

Our genome-wide analyses with population genetics statistics and past demography detected bottleneck events in four Lake Victoria haplochromine species, matumbi hunter, *H. microdon*, *H. chilotes*, and *H. sauvagei*, and we concluded that the upsurge of Nile perch could be the cause of the bottlenecks.

According to the estimated recent demography, population declines began 40 to 30 generations ago (1970s to 1980s) for 4 bottlenecked species [matumbi hunter (f), *H. microdon* (g), *H. chilotes* (h), and *H. sauvagei* (i) in Fig. 3], which is the period when the extinction of endemic haplochromines was becoming a concern (Ogutu-Ohwayo 1990; Kaufman 1992; Witte, Goldschmidt, Goudswaard et al. 1992). After retaining a small population size for 10 to 20 generations, they started to recover the population size 20 to 10 generations ago (1990s to 2000s) and eventually reached the current population size. In fact, in the 2000s, it was reported that the population size of some haplochromines rebounded after the Nile perch expansion (Witte et al. 2007; Natugonza et al. 2021). The timing of the resurgence of the haplochromine population is consistent with the period of bottleneck and quick recovery detected in this study.

Furthermore, we observed multiple signatures of bottleneck events in the 4 species from the comparison of summary statistics. In addition to the earlier discussed demography, the four bottlenecked species showed lower nucleotide diversity *π* and higher inbreeding coefficient *F* among the analyzed 8 species (Fig. 3a and b, and supplementary table S2, Supplementary Material online). The values of SROH and NROH were also mostly high in the 4 bottlenecked species (Fig. 3e). Matumbi hunter and *H. sauvagei* had increased LD coefficients, forming elevated LD decay (Fig. 3c). All these values are typically seen for the population that experienced the bottleneck event.

The timing of the bottleneck in the 4 species corresponded with the period when the number of haplochromines decreased (Fig. 3f to i; Ogutu-Ohwayo 1990; Kaufman 1992; Witte, Goldschmidt, Goudswaard et al. 1992), leaving signs of bottleneck events inferred by multiple summary statistics (Fig. 3a to e). In conclusion, we assume that the Nile perch invasion and the associated population declines could explain bottleneck events in some species.

We expected that piscivores, such as paedophages, would be significantly affected by the Nile perch expansion due to ecological competition (Witte, Goldschmidt, Goudswaard et al. 1992; Seehausen 1996; McGee et al. 2015). This study presented evidence of recent and short-period bottleneck events for 2 paedophages, matumbi hunter and *H. microdon* (Figs. 3f and g). In fact, each species has experienced bottlenecks in similar time periods, suggesting that both species were affected by the Nile perch invasion.

Although recent bottleneck was detected in the 4 species (matumbi hunter, *H. microdon*, *H. chilotes*, and *H. sauvagei*), each species showed slight differences in timing, intensity, and length of a bottleneck (Fig. 3f to i), which are possibly attributed to the differences in habitats, ecologies, and feeding strategies. While matumbi hunter and *H. microdon* are egg eaters, *H. chilotes* and *H. sauvagei* are insect eaters (supplementary table S1, Supplementary Material online). The positive value of the mean Tajima's *D* in *H. chilotes* (0.32, supplementary table S2, Supplementary Material online) can be explained by the current population decline (Fig. 3h). In contrast, even though matumbi hunter, *H. microdon* and *H. sauvagei* underwent bottleneck events, the distribution of Tajima's *D* implied that they are sustaining a constant population size (supplementary fig. S3, Supplementary Material online and supplementary table S2, Supplementary Material online). Genome-wide Tajima's *D* generally reflects the demographic states but is sensitive to the bottleneck's timing, length, and intensity (Schmidt and Pool 2002; Gattepaille et al. 2013). Additionally, we must be aware that the recent population bottleneck, such as the one that occurred less than a few decades ago, is difficult to detect by Tajima's *D* (Ramakrishnan et al. 2005). Therefore, the mean Tajima's *D* calculated in this study possibly reflected the demographic patterns older than the bottleneck caused by the recent expansion of Nile perch.

For *P. nyererei*, *P. pundamilia*, and *L. rufus*, we did not observe any signatures of bottleneck events caused by the Nile perch invasion. These species showed high nucleotide diversity, low inbreeding coefficient, low LD coefficient, and a short and small NROH regions (Fig. 3a to c and e, respectively), which suggested the bottleneck did not occur in those species. Even though the effective population size for *P. nyererei*, *P. pundamilia*, and *L. rufus* has decreased within the last 5 generations (supplementary fig. S4 a to c, Supplementary Material online, respectively), this does not correspond with the period of the Nile perch upsurge. While samples of *L. rufus* used in this study were caught at Kilimo Island and Nyegezi Bay, like the 4 species that experienced bottleneck events after the Nile perch upsurge, this species broadly inhabits the entire parts of Mwanza Gulf from north to the south. Similarly, although *P. nyererei* and *P. pundamilia* analyzed in the current study were caught outside the Mwanza Gulf, both species are broadly distributed around and inside the gulf. Reports describing detailed prey–predator relationships between certain species and Nile perch are unavailable. We could not find co-shared ecomorphology or habitat within either bottlenecked or not-bottlenecked species. A thorough analysis by increasing genomic data in the future would explain why certain species received stronger impacts than others.

### Severe Bottleneck in Matumbi Hunter

Interestingly, the bottleneck event in the matumbi hunter was the most severe among the 4 bottlenecked species. Their minimum effective population size at the decreased phase of the bottleneck was the smallest among any other species (Fig. 3f). They showed the most extreme values in all population genetics statistics computed, such as the lowest nucleotide diversity (Fig. 3a), excess homozygosity as seen in the highest inbreeding coefficient and extended ROH regions (Figs. 3b and e), and elevated LD plots (Fig. 3c).

Matumbi hunter also showed a distinct genetic structure, indicating genetic differentiation from other sympatric species (Fig. 2). However, the phylogenetic analysis supported that matumbi hunter was the ingroup of the paedophage clade (Fig. 4 and supplementary fig. S6, Supplementary Material online). The estimation of speciation time did not indicate early divergence in any pairs of 7 Lake Victoria species including matumbi hunter, in comparison to those between the Lake Victoria species and outgroup riverine species from the Congolese lineage (Fig. 6). Given that genetic drift during the bottleneck can increase the genetic divergence (Chakraborty and Nei 1974, 1977), we assume that the genetic drift during the severe bottleneck eliminated many of alleles inherited from the common ancestor of haplochromines, leading to the high genetic differentiation of matumbi hunter from even sister species.

Alternatively, habitat expansion and the unique phenotypes of the matumbi hunter could also explain the elevated genetic differentiation. The current major habitats of matumbi hunters are the northern part of Mwanza Gulf, such as Kilimo Island and Nyegezi Bay, where samples analyzed in this study were mainly collected (Fig. 1b). However, Seehausen (1996) (survey in 1991 to 1996) initially found matumbi hunter only at Matumbi Island, which is the central part of Mwanza Gulf, although the current major habitats of matumbi hunter were also surveyed. When Mizoiri et al. (2008) carried out a field survey from 2004 to 2005, they found matumbi hunter in the current habitats but not at Matumbi Island, implying that they might have expanded to their current habitats after the initial report by Seehausen (1996). Moreover, the environment where the matumbi hunter dwells varies by location (e.g. they are generally captured in rocky areas at Kilimo Island and Nyegezi Bay but in sandy shallow areas at Nyaruwambu Bay), although cichlids typically inhabit a similar environment irrespective of the location (Mizoiri et al, (2008). The current population size of the matumbi hunter is as large as that in the pre-Nile perch phase, probably because they succeeded in expanding niches during the post-Nile perch era. Matumbi hunter also has some unique phenotypes, such as slender and gray-colored bodies (Fig. 1a), which are hardly observed among either paedophages or rock-dwelling cichlids, and inclined outer teeth in the lower jaws, unlike other paedophages (Seehausen 1996). In addition to genetic drift, the local adaptation and their morphological features and colorations contributed to the matumbi hunter forming the unique genetic structure, possibly contributing to greater negative impacts on their population size from the Nile perch invasion.

### Phylogenetic Origin of Within-lake Trophic Adaptation for Paedophages

This is the first example of large-scale population genomics of paedophages in Lake Victoria, providing the distinct genetic characteristics for the unique trophic group, paedophages.

Consistent with previous phylogenetic studies (Wagner et al. 2013; McGee et al. 2020; Meier et al. 2023), paedophages mostly formed a monophyletic clade in all reconstructed topologies in our phylogenetic analyses using multiple methods (Fig. 4 and supplementary fig. S5, Supplementary Material online). These paedophages are endemic to Lake Victoria and are characterized by distinct morphological features shared within the genus, such as distendable oral jaws, relatively hypertrophic lips, small teeth, and decreased inner tooth rows (Seehausen 1996). The phylogenetic relatedness and morphological similarities of paedophages indicate that paedophages likely originated only once within-Lake Victoria, unlike having multiple origins or migrating from the other basins.

A recent study found that the source of a certain proportion of alleles retained among paedophages, which may be associated with their ecomorphology, was older paedophages from nearby lakes (Meier et al. 2023). In ADMIXTURE, the genetic components exclusively shared among paedophages remained (pale green for *K* = 6 and 7, and purple for *K* = 8 in supplementary fig. S1a, Supplementary Material online), while there are also components shared among the paedophages, *Pundamilia* species, and slightly in other haplochromines, too (supplementary fig. S1a, Supplementary Material online). Future genomics and transcriptomics will discover genes involved in ecological specialization and the molecular basis controlling their unique feeding strategy.

Furthermore, this study supports the close genetic relationship between paedophages and the genus *Pundamilia*. The recently estimated phylogenetic trees using genome data demonstrated the genus–genus level of phylogenetic relationships, revealing the strict monophyly of paedophages and that *Pundamilia* species defined as “*Pundamilia* I” were the sister species of paedophages (McGee et al. 2020; Meier et al. 2023). The networks of genome-wide identity-by-descent (IBD) blocks revealed that paedophages shared a large proportion of IBD blocks with *Pundamilia* I (McGee et al. 2020). Our ADMIXTURE analyses also suggested a shared genetic ancestry between paedophages and *Pundamilia* I, but not with other *Pundamilia* species that do not belong to *Pundamilia* I (Fig. 2a and supplementary fig. S1a, Supplementary Material online). Interestingly, Seehausen (1996) reported *Pundamilia* sp. “nyererei paedophage” from field observation in the western Mwanza Gulf, a newly discovered and undescribed species. Given the close phylogenetic relationship between paedophage and *P. nyererei*, cichlids with such hybrid phenotypes may be sustaining features from both lineages, reflecting a state in the middle of divergence. Species belonging to *Pundamilia* I are either zooplanktivores or insectivores, not paedophages; therefore, alleles differentially fixed among paedophages and *Pundamilia* I would be the key to understanding the trophic adaptation of unique dietary groups.

### Effective Approaches to Overcoming incomplete lineage sorting in Lake Victoria Cichlids

The phylogenetic analyses produced a fine-scale consensus tree, demonstrating the efficacy of estimates in different approaches using genome-wide SNPs from multiple samples per species (Fig. 4 and supplementary fig. S5, Supplementary Material online).

Previous studies built phylogenetic trees from mtDNA, nuclear genes, and the whole genome using a single methodology, basically with a single or few samples per species (Samonte et al. 2007; Wagner et al. 2013; McGee et al. 2020; Meier et al. 2023). Lake Victoria cichlids generally have a low genetic difference due to their young radiation age and recent or ongoing gene flow (Meier, Sousa, et al. 2017), leaving excess polymorphism even within a species and frequently causing incomplete lineage sorting (ILS) (Nikaido et al. 2014; Salzburger 2018). Thus, using multiple samples per species, as in this study, is appropriate to accurately infer the population history of Lake Victoria cichlids rather than the previously taken approach, which used only one species per species.

We performed phylogenetic analyses using multiple approaches to overcome the uncertainty in the topology caused by ILS. In particular, considering the high heterozygosity of haplochromines due to the recent speciation, we used ASTRAL-III, a method known as an effective phylogenetic analysis tool for recently diverged lineages (Zhang et al. 2018). Since it uses phased data as input and considers the assignment of heterozygous sites for phylogenetic estimation, it is theoretically appropriate for Lake Victoria cichlids. Furthermore, ASTRAL-III and SVDquartets, both coalescent-based approaches, showed the same topology except for the position of *P. pundamilia* (Figs. 4a and b). A previous demographic simulation study estimated that gene flow occurred at least twice between *P. pundamilia* and *P. nyererei* from the Makobe populations within the last 6,000 years (Meier, Sousa, et al. 2017). The admixture events likely complicated phylogenetic inferences, causing the varying position of *P. pundamilia* in the constructed phylogenetic trees.

Interestingly, the TWISST topology weighting presented the signals of ILS (Fig. 5b). Topology weighting assumes that the most often seen topology throughout the genome should represent the species tree, even if there is partial gene flow across the investigated species (Martin and Van Belleghem 2017). However, in our topological weighting, although topo1 was observed most frequently among the 15 possible topologies, the difference in average weightings between topo1 and the other 14 topologies was small (Fig. 5b). The 14 topologies were observed in similar frequencies throughout the genome, indicating substantial topological variation by genomic region and a high extent of ILS, as expected.”

### The Inferred Speciation Time Supports the Previously Proposed Scenario for Cichlid Radiation

The estimated speciation times in pairs of investigated species supported the evolutionary scenario in which Lake Victoria haplochromines diverged after the recent and rapid adaptive radiation (Fig. 6). The diversification of haplochromines in Lake Victoria and its surrounding regions began around 100,000 to 200,000 years ago (Verheyen et al. 2003; Seehausen 2006; Genner et al. 2007; Bezault et al. 2011). Although the lake experienced desiccation approximately 15,000 years ago (Johnson et al. 2000), fossil records have revealed that haplochromines have continuously existed in the lake even during the dry period, and those surviving lineages are the ancestors of haplochromine radiation within the lake (Meier et al. 2023; Ngoepe et al. 2023). Nakamura et al. (2021) estimated the speciation time in pairs of *H.* sauvagei- *H. chilotes*, *H.* sauvagei- *L. rufus*, and *H.* chilotes- *L. rufus* as 14,700, 15,600, and 8,800 years ago, respectively. In this study, the average split time of *A. stappersii*, a riverine species, and Lake Victoria haplochromines was 37,421 years ago, and that within the lake was 19,158 years ago, which was relatively older than previous reports (Fig. 6 and supplementary table S3, Supplementary Material online). We should be aware that split-time estimation may be sensitive to the quality of genomes, analyzed species, and SNP filtering options. A previous study using mtDNA and some nuclear genes indicated that *H. sauvagei* diverged 41,300 years earlier than other Lake Victoria cichlids (Samonte et al. 2007; Takeda et al. 2013). However, we did not observe the signature of early divergence in any pair, including *H. sauvagei*. Our estimation does not conflict with the previous geographical events in the lake and corresponds to the estimated period of the adaptive radiation of Lake Victoria haplochromines.

## Conclusion

We successfully demonstrated the population history and phylogenetic relationship of endemic haplochromines by performing large-scale comparative genomics. This study is the first example to present the impacts of the Nile perch upsurge on the genetic structure of Lake Victoria haplochromines. Signatures of bottleneck events in multiple endemic species further supported that the introduction and subsequent expansion of the exotic species Nile perch negatively influenced the demography of endemic species and eventually altered the genetic structure. The previously known hypothesis that piscivores, like paedophages, should have experienced a stronger bottleneck was further supported by inferred evidence of the intense bottleneck in paedophages, especially in matumbi hunters.

## Supplementary Material

msae093_Supplementary_Data
