# Supplementary material for: Severe Bottleneck Impacted the Genomic Structure of Egg-Eating Cichlids in Lake Victoria
Source: Mol Biol Evol. 2024 May 24;41(6):msae093. doi: 10.1093/molbev/msae093 (PMC11166178; doi:10.1093/molbev/msae093)
Supplement: msae093_Supplementary_Data [file msae093_supplementary_data.zip › Imamoto_supplementary_legends_revised.final.pdf]

Legends to supplementary Figures

Figure S1. ADMIXTURE results and Cross-validation (CV) Errors. (a) ADMIXTURE analysis was performed for the *K* = 2 to *K* = 8 range. Nilotic lineage (*Astatotilapia bloyeti*, *A. paludinosa*, *Haplochromis gracilior*, and *Thoracochromis pharyngalis*) and Congolese lineage (*A. stappersii*) are added as an outgroup to Lake Victoria haplochromines. Paedophages, matumbi hunter, *H. microdon*, and *Lipochromis* spp. (*Lip. parvidens*, *Lip. melanopterus*, and *Lip. cryptodon*) have asterisks on each name label, and labels are shaded by a yellow box. Species belong to '*Pundamilia* I,' *Pundamilia nyererei*, and five species are labeled as part of '*Pundamilia* spp.' (*P*. sp. 'big blue red,' *P. igneopinnis*, *P*. sp. 'nyererei-like,' *P*. sp. 'orange,' and *P*. sp. 'pundamilia-like'), are shaded by a light brown box. Labels for all samples are listed in the column 'Label in ADMIXTURE' in Table S1. (b) Cross-validation (CV) error comparison by *K*. Lower CV errors at a specific *K* value indicates more reliable results.

Figure S2. Population structures inferred by PC3-PC4 (with(a) and without outgroups(b)) and PC5-PC6(c). The Nilotic lineage (*Astatotilapia bloyeti*, *A. paludinosa*, *Haplochromis gracilior*, and *Thoracochromis pharyngalis*) and the Congolese lineage (*A. stappersii*) are added as an outgroup to Lake Victoria haplochromines and plotted as square markers. Paedophages, matumbi hunter, *H. microdon*, and *Lipochromis* spp. (*L. parvidens*, *L. melanopterus*, *and L. cryptodon*) have asterisks on each name label and are plotted as triangle markers. 'Paedophages' and '*Pundamilia* I' are plotted as triangle and pentagon markers. Species belonging to '*Pundamilia* I,' except for *P. nyererei*, are plotted as light brown pentagon markers. The contribution rate for each principal component (PC) is indicated on the axis.

Figure S3. The distribution of Tajima's *D* in 10 kb windows across the genome. The range between the first and third quantiles is represented by a thick line in each violine plot, and a white circle indicates the median. Paedophages (matumbi hunter and *Haplochromis microdon*) are shaded by a yellow box with an asterisk on the name label.

Figure S4. The changes in effective population size (N_e) for four not-bottlenecked species ((a) *Pundamilia nyererei*, (b) *P. pundamilia*, (c) *Lithochromis rufus*, and (d) *Astatotilapia stappersii*) during the past 700 generations (years). The GONE estimate was repeated 200 times, and all results were plotted.

Figure S5. DensiTrees constructed by ten un-concatenated datasets for SNAPP with a consensus topology (represented by a ticked blue line). The thinned dataset number is indicated in the corner of each tree. *Astatotilapia burtoni* (Abur), a riverine lineage, was used as the outgroup for all analyses. Samples are labeled with species name abbreviations: Hmat (matumbi hunter), Hmid (*Haplochromis microdon*), Lpar (*Lipochromis parvidens*), Lmel (*Lip. melanopterus*), Lcry (*Lip. cryptodon*), Pnye (*Pundamilia nyererei*), Ppun (*P. pundamilia*), Hchi (*H. chilotes*), Hsau (*H. sauvagei*), and Lruf (*Lithochromis rufus*). Paedophages are indicated with asterisks on the name labels. The more commonly observed trees are colored blue, red, and green, while the remaining trees are shown in dark green.

Legends to supplementary Tables

Table S1. Summary information of all genome samples analyzed in the current study. The ‘Color’ column is shaded by a color representing each species, and species belonging to ‘paedophage’ or ‘*Pundamilia* I’ are labeled. If the exact locality in Lake Victoria where the sample has been caught is known, it was noted in the ‘Locality’ column; otherwise, it was written as ‘unknown.’ For samples newly sequenced in this study and previously sequenced in our group, sampled years are written in the ‘Sampled year’ column. If the sample was included in the 1. Population dataset, 2. Statistics dataset, or 3. Phylogeny dataset marked as ‘+’ (‘-’ if not included) in the ‘ds1’, ‘ds2’, and ‘ds3’ columns, respectively. Bin numbers as x-labels for ADMIXTURE analysis are assigned in the ‘Label in ADMIXTURE’ column. McGee et al. (2020) and Seehausen (1996) defined *Lipochromis cryptodon* and *L.* sp. ‘velvet black cryptodon’ as different species; we summarized them as one species because no elevated genetic differentiation has been detected between them.

Table S2. Summary of population statistics metrics inferring the signatures of a bottleneck. The genome-wide mean values of nucleotide diversity (π), inbreeding coefficient (F), linkage disequilibrium (LD) coefficient (r^2), and the mean and the variance of Tajima’s D, are listed in the table, respectively. Paedophages have an asterisk on the name label. All the statistics were calculated by sample and finally averaged by species. The LD coefficient in the *Haplochromis microdon* was not estimated because a lack of sampling numbers can highly bias the calculation of r^2.

Table S3. Summary of mean split times in 21 pairs for 7 species and average split time among analyzed Haplochromines in Lake Victoria**.** Samples are labeled with species name abbreviations: Asta (*Astatotilapia stappersii*), Hmat (matumbi hunter), Hchi (*Haplochromis chilotes*), Hsau (*H. sauvagei*), Lruf (*Lithochromis rufus*), Pnye (*Pundamilia nyererei*), *and* Ppun (*P. pundamilia*). Paedophages have an asterisk on the name label. The estimation of split time was repeated 100

times per pair. The per-generation mutation rate was set at 3.5×10^{-9}, referred to Malilnsky et al. (2018).
