# Supplementary figures and images for: Severe Bottleneck Impacted the Genomic Structure of Egg-Eating Cichlids in Lake Victoria
Source: Mol Biol Evol. 2024 May 24;41(6):msae093. doi: 10.1093/molbev/msae093 (PMC11166178; doi:10.1093/molbev/msae093)

**Figure S6**

**1.**

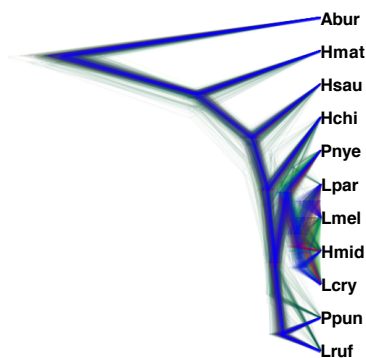

**2.**

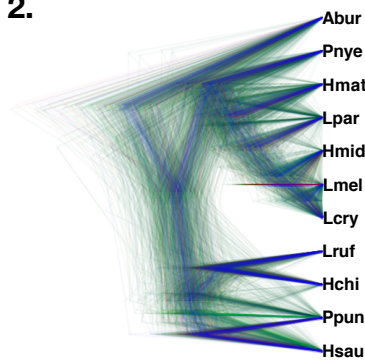

**3.**

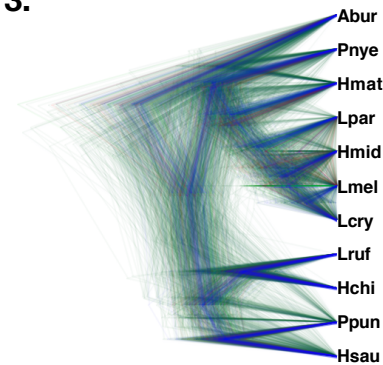

**4.**

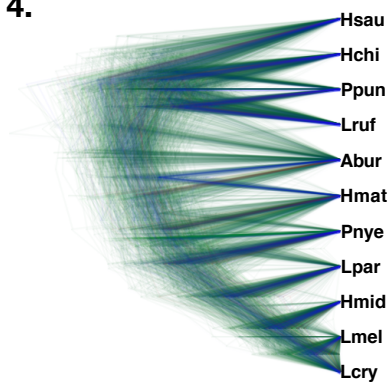

**5.**

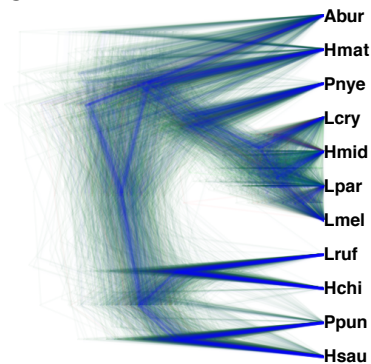

**6.**

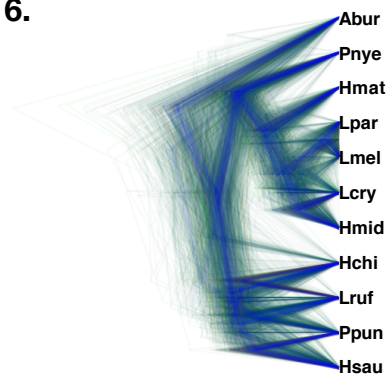

**7.**

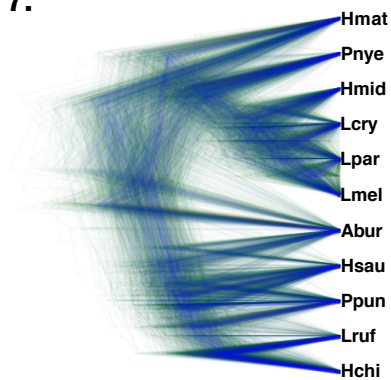

**8.**

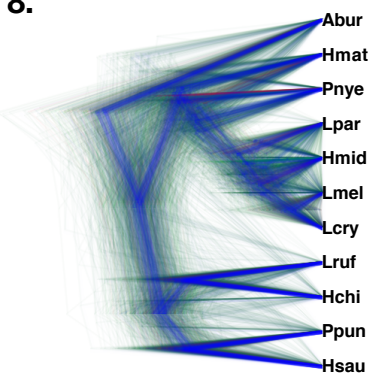

**9.**

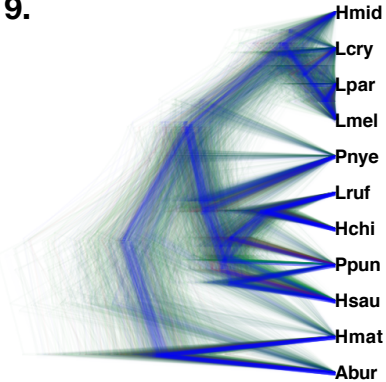

**10.**

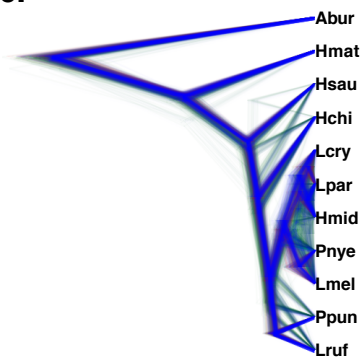

Supplement: msae093_Supplementary_Data [file msae093_supplementary_data.zip › Imamoto_supplementary_figure6.pdf]

Figure S1

(a)

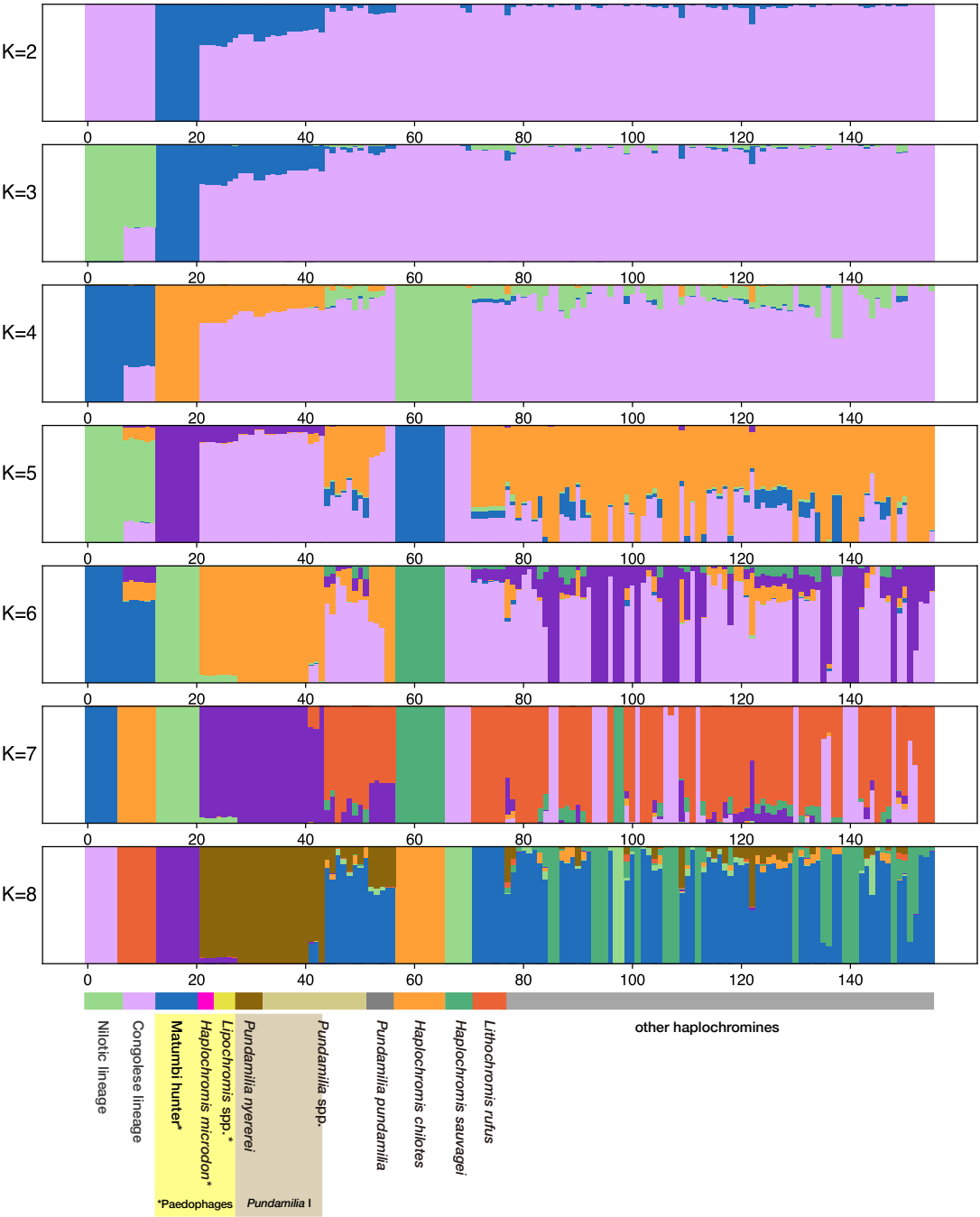

(b)

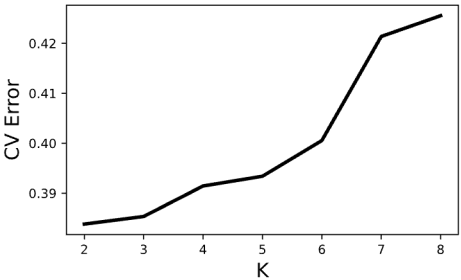

# Figure S2

(a)

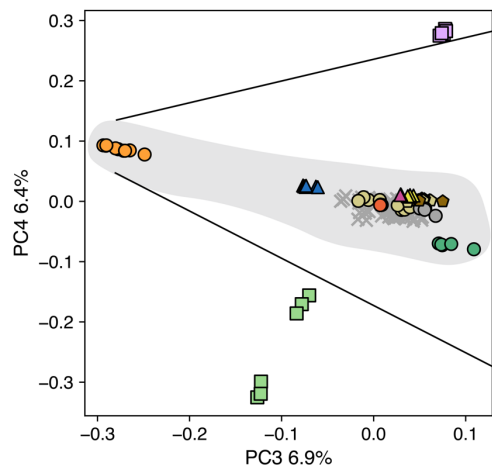

(b)

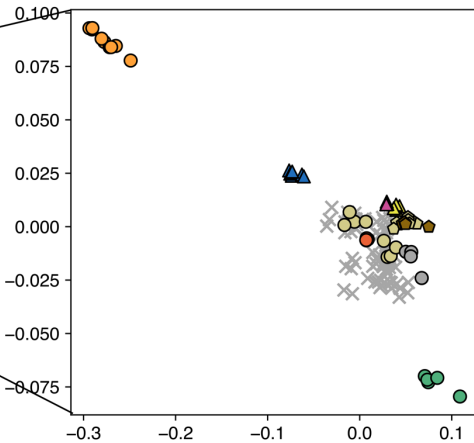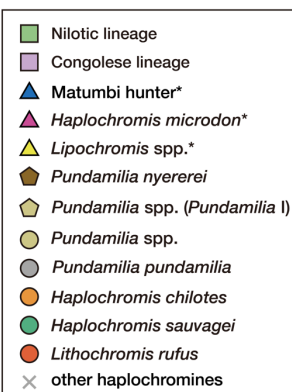

(c)

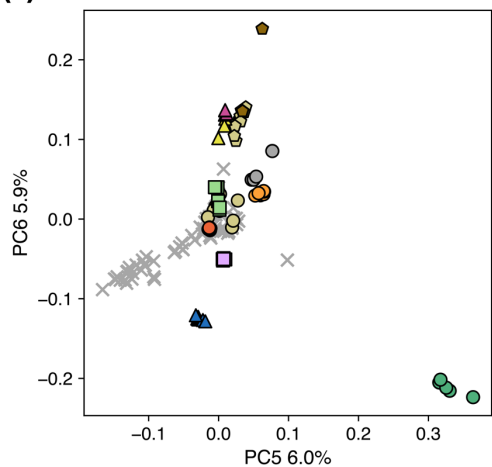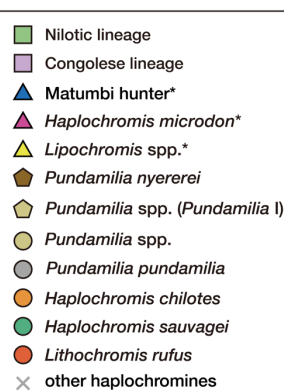

**Figure S3**

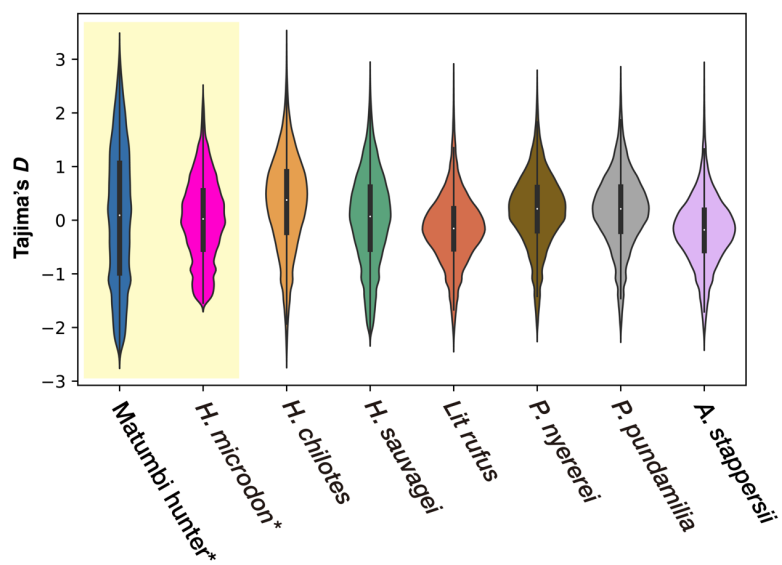

**Figure S4**

**(a)**

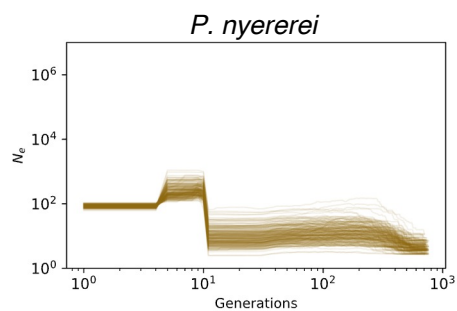

**(b)**

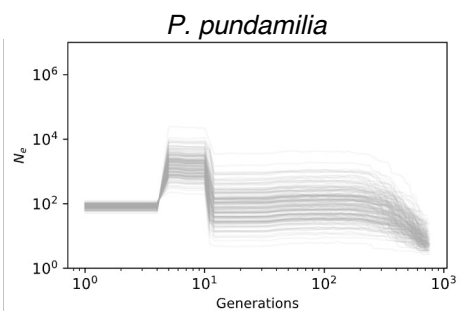

**(c)**

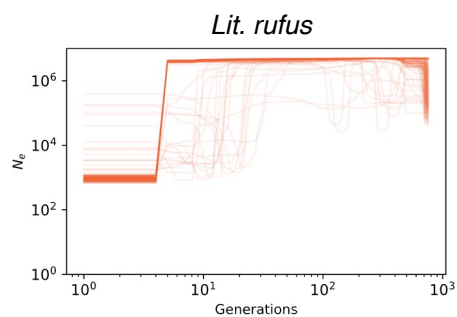

**(d)**

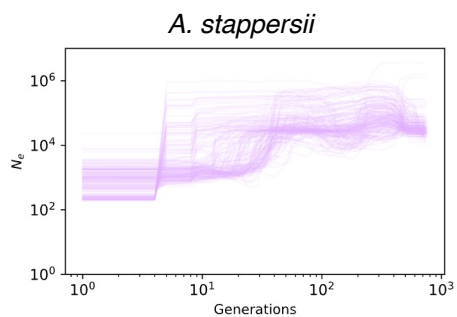

Figure S5

1.

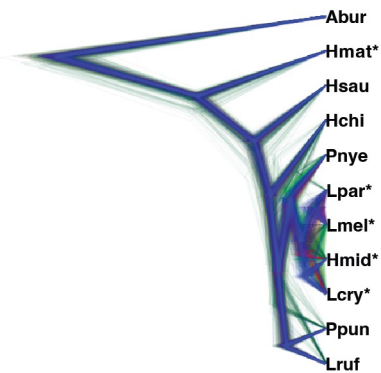

2.

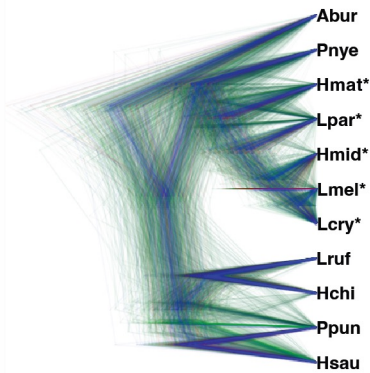

3.

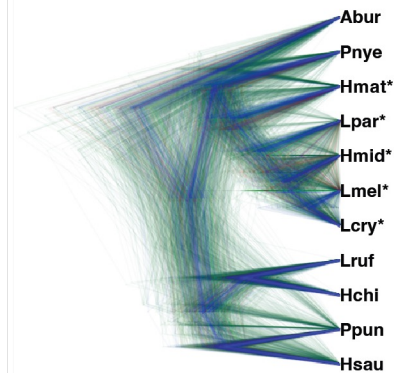

4.

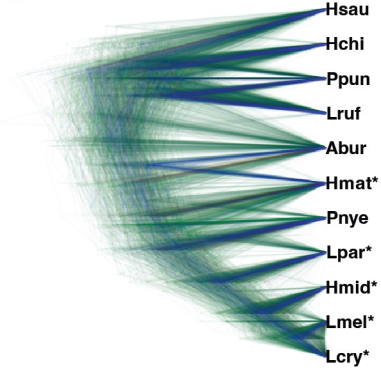

5.

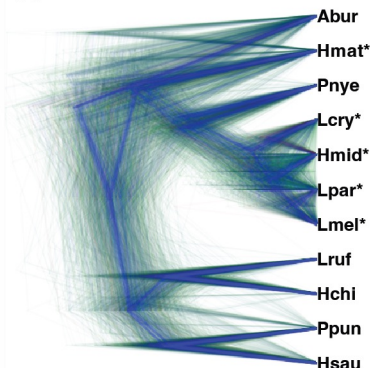

6.

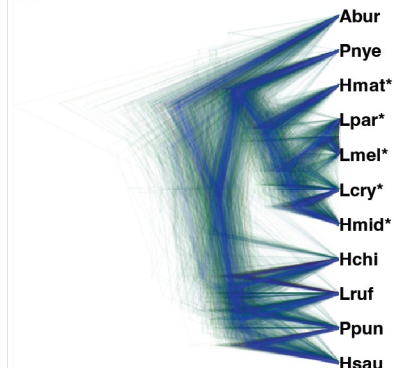

7.

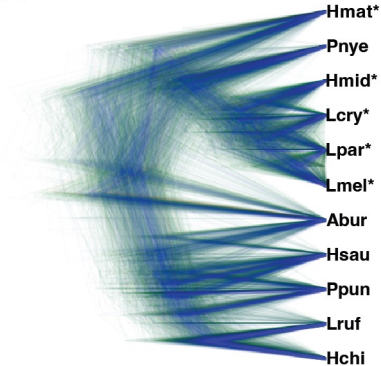

8.

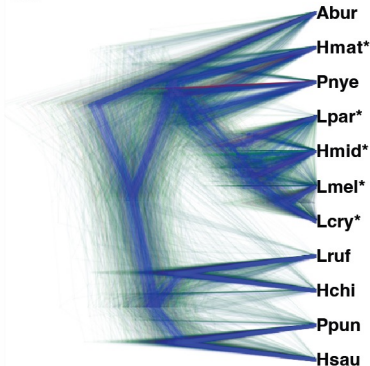

9.

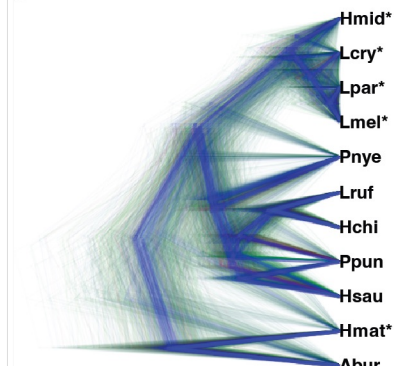

10.

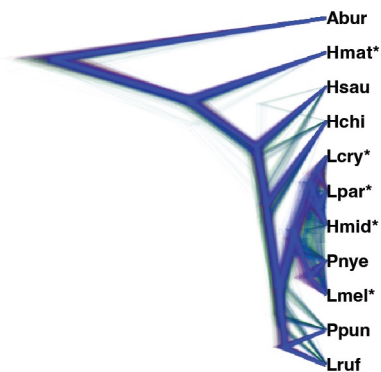

Supplement: msae093_Supplementary_Data [file msae093_supplementary_data.zip › Imamoto_supplementary_figures_revised.final.pdf]
